# Ultraviolet-B Resonant-Cavity Light-Emitting Diodes with Tunnel Junctions and Dielectric Mirrors

**DOI:** 10.1021/acsphotonics.4c00312

**Published:** 2024-07-11

**Authors:** Estrella Torres, Joachim Ciers, Michael A. Bergmann, Jakob Höpfner, Sarina Graupeter, Massimo Grigoletto, Martin Guttmann, Tim Kolbe, Tim Wernicke, Michael Kneissl, Åsa Haglund

**Affiliations:** † Department of Microtechnology and Nanosciense, Chalmers University of Technology, 41296 Gothenburg, Sweden; ‡ Institute of Solid State Physics, Technische Universität Berlin, 10623 Berlin, Germany; § Ferdinand-Braun-Institut (FBH), 12489 Berlin, Germany

**Keywords:** ultraviolet, AlGaN, resonant-cavity light-emitting diode, electrochemical etching, tunnel junction

## Abstract

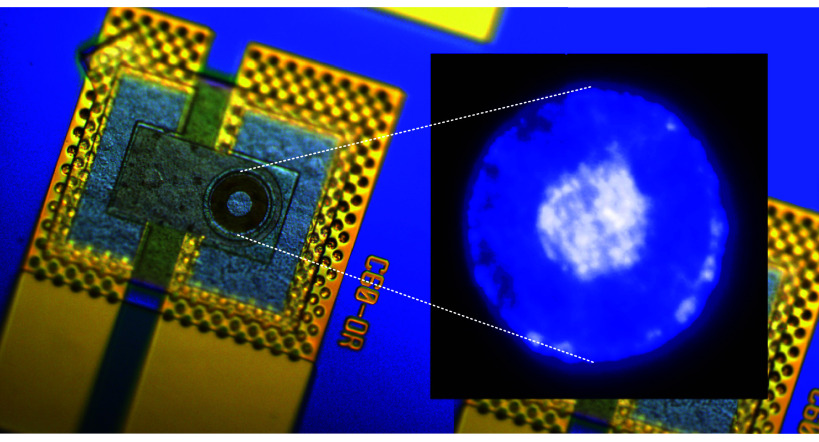

We demonstrate the first electrically injected AlGaN-based ultraviolet-B resonant-cavity light-emitting diode (RCLED). The devices feature dielectric SiO_2_/HfO_2_ distributed Bragg reflectors enabled by tunnel junctions (TJs) for lateral current spreading. A highly doped n^++^-AlGaN/n^++^-GaN/p^++^-AlGaN TJ and a top n-AlGaN current spreading layer are used as transparent contacts, resulting in a good current spreading up to an active region mesa diameter of 120 μm. To access the N-face side of the device, the substrate is removed by electrochemically etching a sacrificial n-AlGaN layer, leading to a smooth underetched surface without evident parasitic etching in the n- and n^++^-doped layers of the device. The RCLEDs show a narrow emission spectrum with a full width at half-maximum (FWHM) of 4.3 nm compared to 9.4 nm for an ordinary LED and a more directional emission pattern with an angular FWHM of 52° for the resonance at 310 nm in comparison to ∼126° for an LED. Additionally, the RCLEDs show a much more stable emission spectrum with temperature with a red-shift of the electroluminescence peak of about ∼18 pm/K and a negligible change of the FWHM compared to LEDs, which shift ∼30 pm/K and show spectrum broadening with temperature. The demonstration of those devices, where a highly reflective mirror is spatially separated from an ohmic metal contact, opens up a new design space to potentially increase the poor light extraction efficiency in UV LEDs and is an important step toward electrically injected UV vertical-cavity surface-emitting lasers.

AlGaN-based ultraviolet (UV) light-emitting diodes (LEDs) have a wide range of applications in the disinfection of air, water, medical tools, and food, UV curing, sensing, skin treatment, greenhouse lighting, and wireless communication.^1^ These applications could benefit from the advantages of resonant-cavity LEDs (RCLEDs), such as a spectral narrowing, a more directional far-field emission pattern, and a reduction of the wavelength shift with temperature and current.^2^

The RCLED design rules suggest that the bottom mirror of the optical cavity should be highly reflective (above 99%) at the targeted wavelength to enhance the resonant effect and minimize the outcoupling of light through the bottom mirror.^2^ In III-nitride-based RCLEDs, which have mainly been explored in the visible wavelength region, the optical cavities have been defined by a combination of metal mirrors,^3^ semiconductor/air interface,^4^ and an epitaxial and/or dielectric distributed Bragg reflector (DBR).^5^ Metallic mirrors are an interesting option due to the combination of electrical injection and reflection. However, in UV, metallic mirrors are strongly absorptive, and highly reflective metallic mirrors are not possible to achieve. For example, UV LEDs with reflective p-contacts, such as Ni/Al,^6^ indium–tin-oxide (ITO)/Al,^7^ and Mo/Al,^8^ on p-AlGaN structures have shown maximum reflectivity values in the range of ∼80 to 87% for UVB, which is low for a bottom mirror when a high quality factor is desired. Furthermore, the contact resistance and device operation voltage are much higher compared to UV-LEDs employing p-GaN contact layers.^9^

Up to date, a few RCLEDs have been demonstrated and only in the UVA^10−12^ by employing porous/airgap DBRs. Many different approaches are being explored in parallel to achieve a more highly reflective structure. Epitaxial UVA AlGaN-based DBRs have shown reflectivity values higher than 99%.^13,14^ However, in the UVB and UVC, the maximum reflectivity achieved so far is 97.7% at 273 nm using 25 pairs.^15^ This is due to the small refractive index contrast and substantial lattice mismatch of AlN/AlGaN layers making the demonstration of AlGaN DBRs above 99% reflectance difficult at these wavelengths. Another approach is a porous DBR in which the refractive index contrast is tuned by porosifying alternating n-AlGaN layers. Reflectivity values up to 93% at 374 nm^12^ and 276 nm^16^ have been achieved with a 12-pair and 20-pair porous DBR, respectively. However, the light scattering at the pores and the potential difficulty in controlling the pore size limits the maximum achievable reflectivity, and mechanical and postprocessing DBR stability could be problematic. Alternatively, dielectric SiO_2_/HfO_2_ DBRs which require a low number of pairs to achieve a high reflectivity, have been used to demonstrate UVB^17^ and UVC^18^ vertical-cavity surface-emitting lasers (VCSELs). The former used a 10-pair dielectric DBR with a measured peak reflectivity of 99.23% at 320 nm, while the latter used a 15.5-pair DBR with 97.7% reflectivity at 276 nm. Recently, dielectric SiO_2_/Ta_2_O_5_ DBRs have also shown reflectivities above 99% at 310 nm.^19^ However, the implementation of all-dielectric DBRs requires substrate removal techniques to access the bottom surface and an electrical injection scheme that allows the electrical contacts to be placed in the periphery of the mesa.

Electrochemical etching is a substrate removal based on a tunneling process carried out at the semiconductor/electrolyte junction, where a sacrificial layer is etched, releasing the device membrane from the substrate.^20,21^ The etch selectivity in this process is determined by the semiconductor bandgaps, n-doping concentrations, and the applied voltage, favoring the etching of more heavily doped layers with a lower bandgap.^21^ This technology has been proven compatible with doped devices such as UVB-LEDs,^22^ providing smoothly etched N-face surfaces and good cavity length control,^17,23^ which are important features for the fabrication of microcavities. To our knowledge, the compatibility of electrochemical etching with devices containing heavily doped n-AlGaN structures, such as tunnel junctions (TJs), has not been proven up until now.

The implementation of dielectric DBRs requires an efficient electrical injection, including a transverse spreading of holes, which is difficult to achieve in UVB devices due to the low p-type conductivity in the UVB-transparent p-AlGaN. The limited hole conductivity is due to the large effective hole mass and Mg acceptor ionization energy. Both of these factors increase with Al molar fraction, reducing p-type conductivity. One approach would be to separate the electrical injection, i.e., the electrical contacts, from the highly reflective structure. For instance, in the visible, this is done with a transparent conductive oxide layer such as ITO on top of p-GaN. Since the ITO spreads the current laterally over the device mesa, p-side contacts can be deposited on the periphery of the mesa, allowing to place a highly reflective mirror in the center region.^24^ However, ITO is strongly absorbent in the UVB-UVC (0.2 to 1.4 × 10^5^ cm^–1^) and, thereby, is not suitable for UVB microcavities. A different concept that allows for the separation of the contacts from highly reflective structures and that can be applied in the UV without much absorption penalty is a combination of a reverse biased heavily doped n^++^-AlGaN/p^++^-AlGaN TJ with a top n-AlGaN current spreading layer, which has been used for the fabrication of UV TJ-LEDs.^25−27^ K. Nagata et al. have reported promising n^++^-Al_0.60_Ga_0.40_N/p^++^-Al_0.60_Ga_0.40_N homojunction TJs with an operating voltage of 8.8 V at 63 A·cm^–2^ by optimizing the thickness of the TJ and the doping concentration.^25^ Inserting a thin interlayer with a lower bandgap such as InGaN^26^ or GaN^27^ could further decrease the operation voltage but lead to an increased optical absorption loss. The former reported an operating voltage of 6.8 V at 10 A/cm^2^ with a graded TJ structure from Al_0.45_Ga_0.55_N (Al_0.55_Ga_0.45_N) to Al_0.55_Ga_0.45_N (Al_0.45_Ga_0.55_N) on the n^++^- (p^++^-) side, while the latter is 21 V at 60 A/cm^2^ with a p^++^-Al_0.75_Ga_0.25_N/n-GaN/n^++^-Al_0.65_Ga_0.35_N TJ. In addition, TJs have been successfully employed for current injection in InGaN-based blue VCSELs.^28,29^

In this work, we fabricated UVB RCLEDs with TJs and all-dielectric DBRs. The devices were defined by a circular active region mesa and a rectangular device mesa. The active region mesa with diameters of 30 μm, 60 μm, 120 μm, and 160 μm, includes a transparent UVB LED structure with a TJ and an n-Al_0.42_Ga_0.58_N current spreading layer on top, see Figure 1a. Two different TJs were investigated consisting of a p^++^-Al_0.35_Ga_0.65_N/n^++^-Al_0.42_Ga_0.58_N stack and a p^++^-Al_0.35_Ga_0.65_N/n^++^-GaN/n^++^-Al_0.42_Ga_0.58_N stack with a 4 nm n^++^-GaN interlayer. The device mesa included the bottom UVB LED n-Al_0.42_Ga_0.58_N layer and a stack of layers for lift-off, where a 4 nm/4 nm n^+^-Al_0.11_Ga_0.89_N/n^+^-Al_0.37_Ga_0.63_N multilayer combined with a n^+^-Al_0.37_Ga_0.63_N bulk sacrificial layer were embedded between two etch block layers. The p- and n-side metal contacts were fabricated with the same V/Al/Ni/Au metal stack due to the TJ. The combination of the reverse-biased AlGaN TJ with the top n-Al_0.42_Ga_0.58_N layer allowed the p-side metal contact to be placed at the circumference, and hence, a highly reflective 12-pair SiO_2_/HfO_2_ p-side DBR could be deposited in the center on top of the active region mesa, see Figure 1b. The substrate was removed by electrochemically etching the sacrificial layer. Subsequently, the device membranes were bonded onto a carrier chip with predefined Au metal pads. Lastly, a 2-pair SiO_2_/HfO_2_ n-side DBR was sputtered, as is illustrated in Figure 1c. The UVB RCLEDs were characterized at three different stages in the process: after p- and n-side contact evaporation (LED), after the bonding of the devices before the n-side DBR deposition (air-RCLED), and after the n-side DBR, i.e., fully processed devices (DBR-RCLED). Further details about the epitaxial growth, device fabrication, and characterization are found in the Methods section in the Supporting Information.

**Figure 1:**
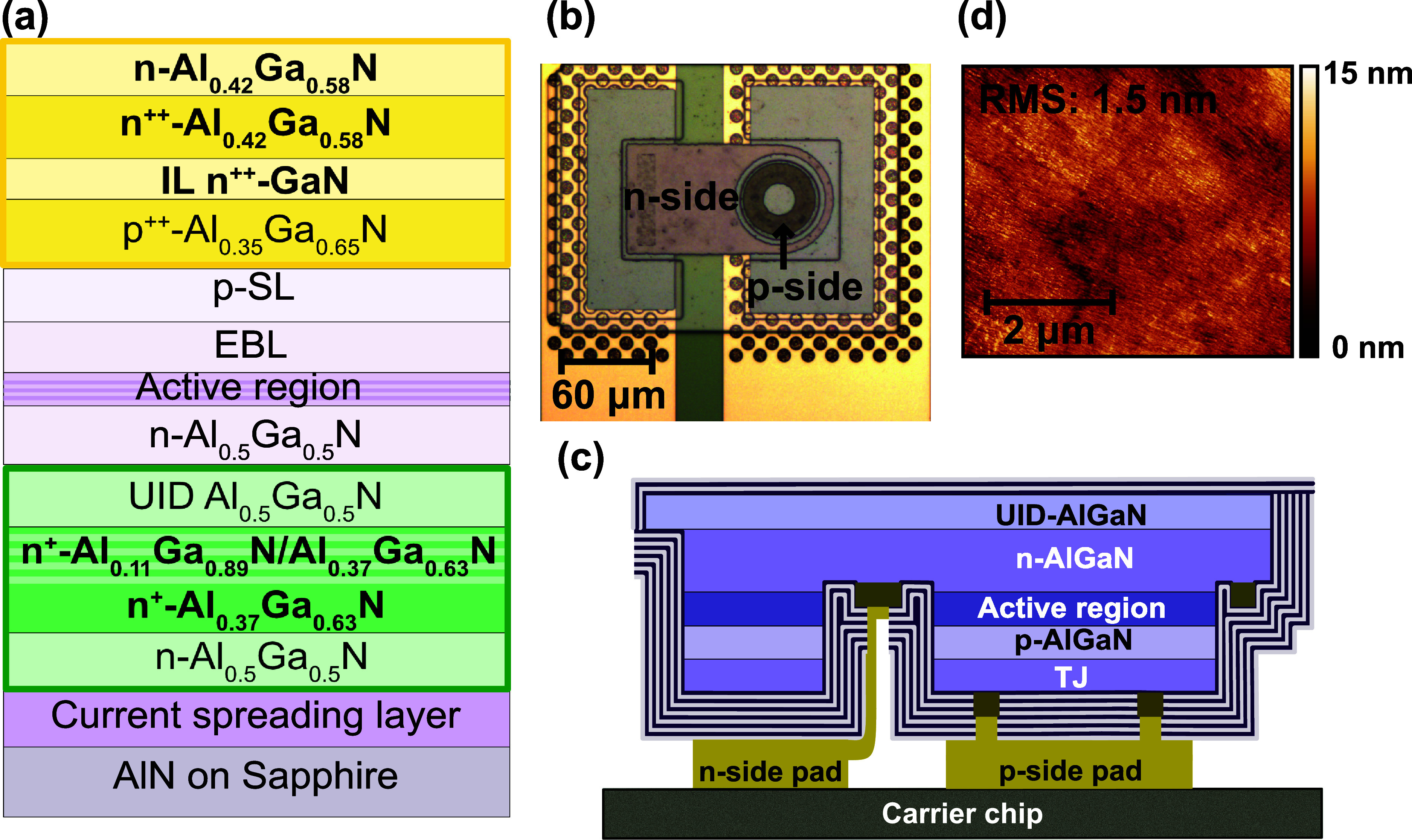
(a) Schematic of the as-grown epitaxial TJ UVB LED structure. The TJ and top current spreader layer are represented in yellow and the layers for electrochemical lift-off are in green. (b) Top view optical microscope image of a flip-chip RCLED with a mesa diameter of 60 μm and a doughnut-shaped p-side contact. (c) Cross-sectional sketch of a DBR-RCLED. (d) AFM image of the under-etched and thereby exposed N-face UID-Al_0.50_Ga_0.50_N surface.

Once the membranes were transferred and flip-chip bonded to the carrier chip, the exposed N-face surface could be evaluated. The LED membranes, including the highly doped TJs, did not show any parasitic etching, indicating effective protection by the p-side DBR, photoresist, and the epitaxially grown etch block layer during the electrochemical etching. Atomic force microscope (AFM) measurements yielded a root-mean-square (RMS) roughness of 1.5 nm over a 5 × 5 μm^2^ area of the N-face surface on top of the circular active region mesa, as illustrated in Figure 1d. The smooth surface is attributed to the built-in polarization fields generated at the interface between UID-Al_0.50_Ga_0.50_N etch block and the n^+^-Al_0.11_Ga_0.89_N/n^+^-Al_0.37_Ga_0.63_N superlattice sacrificial layer which cause an abrupt etch stop.^30^

The spatial distribution of the emission intensity of the DBR-RCLED devices, which shows the horizontal current spreading and the p-side reflectivity, was mapped for different active region mesa sizes at a current density of 30 A/cm^2^, as shown in Figure 2. The emission distribution of the RCLEDs can be separated into the lower intensity circumference delimited by the dashed lines where the p-side contact is placed and the higher intensity center above the p-side DBR where the emission intensity is higher by about 50%. The reason for this higher intensity in the center of the mesa is the highly reflective p-side SiO_2_/HfO_2_ DBR (98.3% reflectivity at 310 nm), to be compared to the region with the low-reflectivity doughnut-shaped p-side contact (27.7% at 310 nm for this annealed V-based contact). Both the DBR and the p-side contact were deposited on a double-side-polished transparent sapphire substrate for reflectivity measurements. No major differences were found in the electro-optical characterization between devices with and without n^++^-GaN interlayer TJ design, see Supporting Information, Figure S2a and b

**Figure 2:**
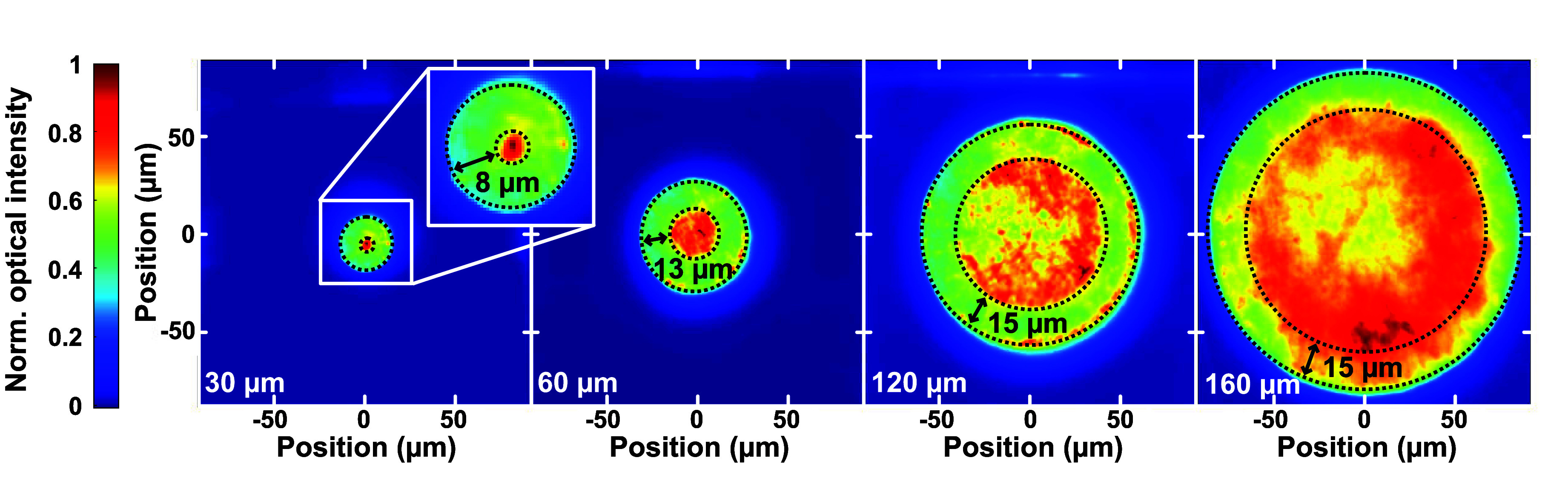
Normalized near-field emission pattern of the DBR-RCLED devices with different active region mesa diameters (left to right: 30, 60, 120, and 160 μm) driven at 30 A/cm^2^. The area delimited by the dashed lines corresponds to the border of the p-side contacts.

For the 160 μm diameter mesa, the current spreading in the top n-AlGaN layer above the TJ is not sufficient and current crowding near the edge of the p-contact is evident. This could be caused by an incomplete activation of the Mg through the mesa sidewalls^31^ or a limited conductivity in the top n-AlGaN current spreading layer. An incomplete activation of Mg results in a lower hole concentration in the center of the mesa than that at the edge. However, this was ruled out by first studying the emission intensity distribution at a low current density (5 A/cm^2^), which was homogeneous. Second, devices with a center disk-shaped p-side contact instead of the doughnut-shaped contact (Supporting Information, Figure S3) show higher intensity close to the center of the mesa, indicating an effective activation of the Mg acceptors. Hence, the increased current crowding for larger mesa sizes is instead ascribed to the high sheet resistance of the top n-AlGaN current spreading (estimated to 1815 Ω/sq by circular transfer length measurements), which is 3.2 times higher than the measured sheet resistance for the n-Al_0.50_Ga_0.50_N layer on the n-side of the LED.

Figure 3a shows a comparison of the electroluminescence (EL) spectrum of the device driven at 120 A/cm^2^ at the LED and the DBR-RCLED stage, where the emission originating above the p-side contacts has been removed from the DBR-RCLED by filtering the spatially resolved spectrum. The LED spectrum shows a broad spontaneous emission peak at 310.3 nm. Thereby, the spontaneous emission couples effectively into the resonant cavity modes in the DBR-RCLED showing two main resonances at 305.3 and 310.6 nm. This results in a spectrally narrowed emission for the DBR-RCLED with a full width at half-maximum (FWHM) of 4.3 nm for the resonance at 310.6 nm instead of 9.4 nm for the LED. The cavity quality factor and hence the FWHM could be limited by the rough as-grown surface; see in Supporting Information, Figure S2c and d.

**Figure 3:**
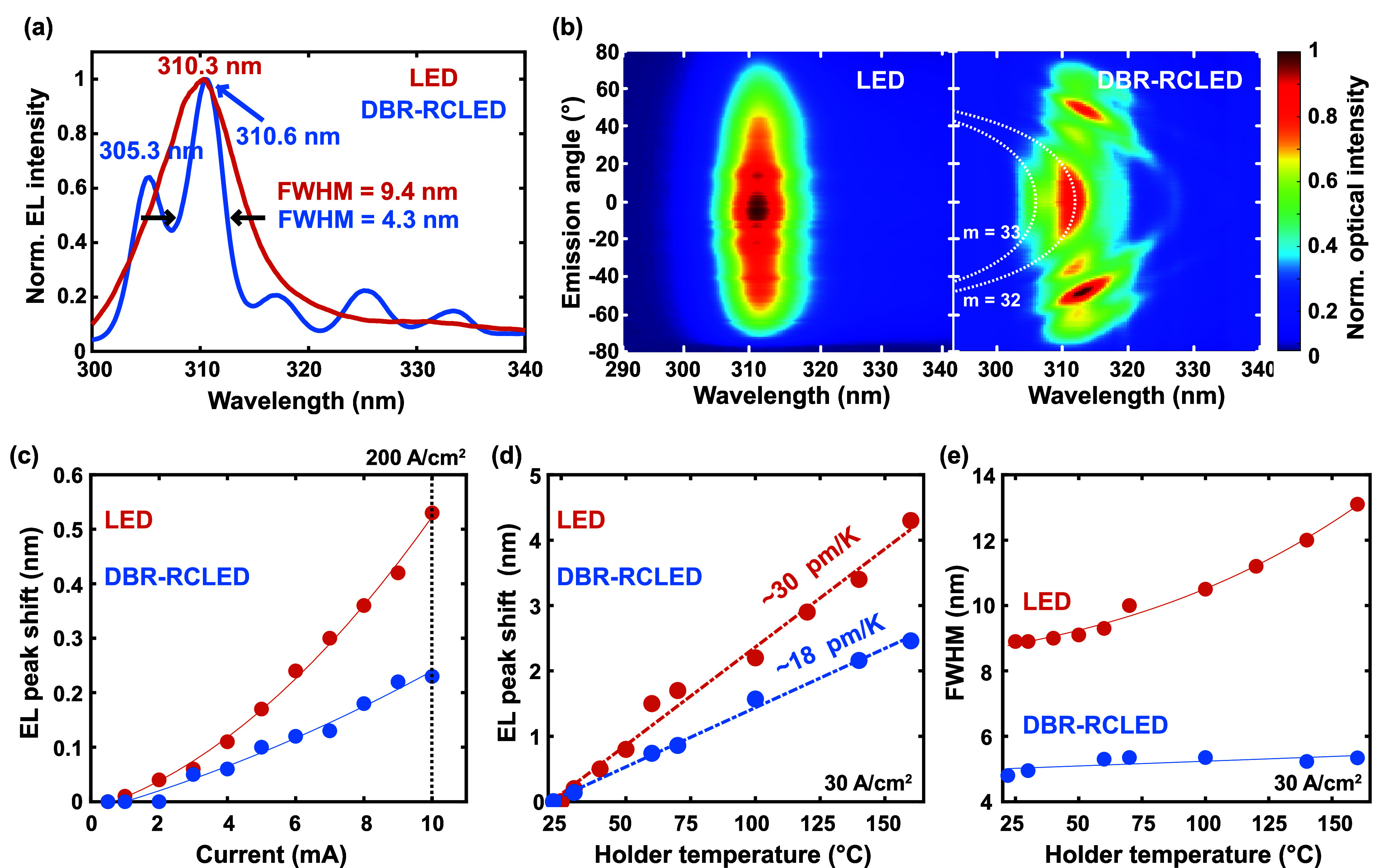
Comparison between devices at the LED and DBR-RCLED stages. (a) EL spectra integrated over a range of 40° at 120 A/cm^2^. The emission originating from the p-side contact area is excluded from the DBR-RCLED spectrum. (b) Angular-resolved EL showing the spectrally resolved far-field spectra of the device at the LED and DBR-RCLED stage at ∼200 and ∼300 A/cm^2^, respectively. The LED has an angular FWHM of ∼126°, and the white dashed parabolas indicate the cavity modes of order 32 and 33 which have an angular FWHM of 52° and 22°, respectively. EL peak shift as a function of (c) current and (d) temperature under a continuous current injection of 30 A/cm^2^. Linear fits in (d) are shown in dashed lines. (e) Temperature dependence of the FWHM at 30 A/cm^2^.

Angle-resolved EL measurements were made to investigate the spectrally resolved far-field emission pattern of the DBR-RCLEDs. Due to the low sensitivity of the far-field setup, higher current densities around 200 and 300 A/cm^2^ at the LED and DBR-RCLED stage were applied, respectively. Figure 3b shows the nondispersive spontaneous emission of the device at the LED stage, with an angular FWHM of ∼126°, which accords with previous values found in the literature.^32,33^ On the other hand, the far-field emission pattern of the DBR-RCLED has parabolically dispersed cavity modes with an angular FWHM of 22° for the 305.3 nm resonance and 52° for the 310.6 nm resonance. This shows the potential of RCLEDs for beam shaping without the need for particular encapsulation approaches. A further improvement in angular beam profile could be achieved by reducing the cavity length that is ∼17λ in these devices, thus increasing the spectral mode spacing, and avoiding multiple modes to overlap with the spontaneous emission of the multi-quantum wells (MQWs).

Current and temperature-dependent EL measurements were done in devices at the LED and DBR-RCLED stage. Figure 3c shows that the EL peak of the LED red-shifts more than the main resonance of the DBR-RCLED when the devices are driven to 200 A/cm^2^. The red-shift of the LED’s EL peak is a consequence of Joule heating in the device leading to an increase in the junction temperature, and hence a lowering of the bandgap of the active region. While this effect is directly seen in the LED spectrum, the DBR-RCLED’s EL peak red-shift is a consequence of the refractive index and physical cavity length variation with temperature, which is smaller than the bandgap reduction with the temperature. To get a direct measure of how much the emission spectra change with temperature, temperature-dependent EL measurements were performed at a current density of 30 A/cm^2^. Figure 3d shows the red-shift of EL peak for the device at the LED and DBR-RCLED stage when the devices are heated up from room temperature to 160 °C. The wavelength increases linearly with temperature with a slope of ∼30 pm/K for the LED and ∼18 pm/K for the DBR-RCLED. While the spectrum of the LED broadens with temperature from 8.9 to 13.1 nm, the change in FWHM of the main resonance of the DBR-RCLED is negligible, see Figure 3e. The red-shift of the EL peak with temperature for the LED is in good agreement with previously reported values of UVB active regions,^34^ and the red-shift of the main resonance in the DBR-RCLED is in good agreement with that of GaN-based cavities.^24^

Figure 4 shows a comparison of two RCLEDs with different quality factors, i.e., the air-RCLED and the DBR-RCLED. The cavity of the air-RCLEDs is defined by the bottom DBR and AlGaN/air interface with reflectivity values of 98.3% and 18.2% at 310 nm, respectively. Once the 2-pair DBR is sputtered on the n-side to fabricate the DBR-RCLED, the reflectivity at the top interface increases to 61.3% at 310 nm. Figure 4a shows the spectrally resolved far-field values for both devices. The parabolic dispersion of the cavity modes is visible in both cases; the spectral FWHM is 4.3 nm for the DBR-RCLED and 6.6 nm for the air-RCLED (Figure 4b). This is ascribed to the enhancement of the quality factor by increasing the reflectivity of the top surface. The light-current–voltage (L-I-V) characteristics of both devices show a turn-on voltage of 5.22 V and negligible degradation of the I-V by the sputtered top DBR (Figure 4c). However, the optical power at 120 A/cm^2^ decreases by ∼35% when the top interface has a higher reflectivity. This is attributed to the enhancement of light confinement in the cavity increasing the probability of reabsorption in the active region and hence decreasing the total optical output power. This comparison highlights the trade-off between spectral purity and total optical power.

**Figure 4:**
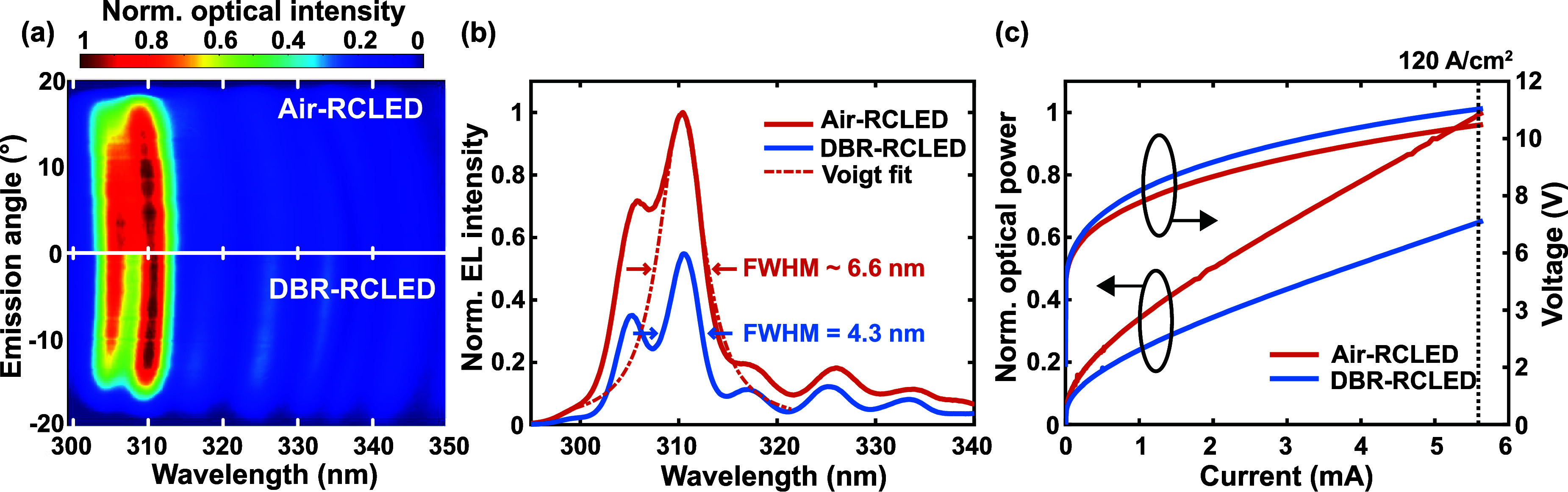
Electro-optical comparison of an air-RCLED (without n-side DBR) with a DBR-RCLED: (a) angular-resolved EL, (b) EL spectra integrated over all angles, including a Voigt fit at 120 A/cm^2^, and (c) L-I-V characteristics of the devices driven up to 120 A/cm^2^.

In summary, we fabricated the first electrically injected UVB RCLEDs. The devices were enabled by UV-transparent contacts, including TJs and a top n-AlGaN current spreading layer, using electrochemical etching for substrate removal to facilitate the use of all-dielectric DBRs. This shows the compatibility of underetching devices with heavily doped layers without parasitic electrochemical etching when they are properly isolated from the electrolyte. Moreover, the UV-transparent contacts provide a good current spreading for mesas with a diameter up to 120 μm which allows the integration of highly reflective mirrors, independently from the p-side metal contacts. Additionally, the DBR-RCLEDs show a 46% narrower spectral emission and a more directional emission pattern (FWHM ∼52° for the 310.6 nm resonance). In addition, the RCLED shows a more stable EL spectrum with current and temperature. The main resonance shifts by ∼18 pm/K in contrast to the ∼30 pm/K shift for the LED and the FWHM experiences a negligible change while the LED’s FWHM broadens from 8.9 nm at room temperature to 13.1 nm at 160 °C. There is a trade-off between the spectral narrowing and the total optical power of the RCLEDs. Future work should be focused on reducing the cavity length to avoid multiple cavity modes overlapping with the spontaneous emission of the MQWs. In addition, optimization of the as-grown surface and the reflectivity of dielectric DBRs are necessary to reduce mirror losses. Lastly, the emission originating above the p-side contacts could be avoided by including a current confinement aperture. All these considerations are important steps toward higher-performing RCLEDs and the first demonstration of electrically injected UV VCSELs.

## Supporting Information Available

Details on the epitaxial growth, device fabrication, characterization setup, the performance of RCLEDs with different TJ designs, and RCLEDs with a center disk-shaped p-contact (PDF)
