# Supplementary material for: Ultraviolet-B Resonant-Cavity Light-Emitting Diodes with Tunnel Junctions and Dielectric Mirrors
Source: ACS Photonics. 2024 Jul 11;11(8):2923–9. doi: 10.1021/acsphotonics.4c00312 (PMC11342380; doi:10.1021/acsphotonics.4c00312)
Supplement: Supplementary file 1 — ph4c00312_si_001.pdf [file ph4c00312_si_001.pdf]

Supporting Information - Ultraviolet-B resonant-cavity light-emitting diodes with tunnel junctions and dielectric mirrors

Estrella Torres,[*,†] Joachim Ciers,[†] Michael A. Bergmann,[†] Sarina Graupeter,[‡] Massimo Grigoletto,[‡] Jakob Höpner,[‡] Martin Guttmann,[¶] Tim Kolbe,[¶] Tim Wernicke,[‡] Michael Kneissl,[‡,¶] and Åsa Haglund[†]

†*Department of Microtechnology and Nanosciense, Chalmers University of Technology, 41296 Gothenburg, Sweden*

‡*Institute of Solid State Physics, Technische Universität Berlin, 10623 Berlin, Germany*

¶*Ferdinand-Braun-Institut (FBH), 12489 Berlin, Germany*

E-mail: estrella@chalmers.se

Methods

Epitaxial growth

The epitaxial structure was grown by metal-organic vapor phase epitaxy using a 3x2 inch close-coupled shower head reactor on an AlN/sapphire pseudosubstrate in the [0001] direction.[1] The buffer layer starts with a GaN/AlN super-lattice for stress management followed by a layer graded from $Al_{0.72}Ga_{0.28}N$ to $Al_{0.55}Ga_{0.45}N$. The growth continued with a stack of layers for electrochemical lift-off which consists of an n-$Al_{0.50}Ga_{0.50}N$ lateral current spreading layer with a Si concentration of $3x10^{18}$ cm^{-3} followed by an n-$Al_{0.50}Ga_{0.50}N$ and unintentionally doped-$Al_{0.50}Ga_{0.50}N$ etch block layers embedding a sacrificial layer.[2] To allow lower Al concentrations without compromising the crystal quality of the structure and smoother N-face surfaces,[3] a multilayered sacrificial layer formed by a 95 nm n^+-$Al_{0.37}Ga_{0.63}N$ and 4 nm/4 nm n^+-$Al_{0.11}Ga_{0.89}N$/n^+-$Al_{0.37}Ga_{0.63}N$ super-lattice both with a Si doping concentration of $2x10^{19}$ cm^{-3} was chosen.[4] Subsequently, the ultraviolet-B (UVB) light-emitting diode (LED) heterostructure with a targeted wavelength at 310 nm was grown, consisting of a 1 μm thick n-$Al_{0.50}Ga_{0.50}N$ layer, a threefold $Al_{0.21}Ga_{0.79}N$/$Al_{0.33}Ga_{0.67}N$ multi-quantum wells active region, a p-$Al_{0.80}Ga_{0.20}N$ electron blocking layer[5] and a heavily doped p^{++}-$Al_{0.35}Ga_{0.65}N$ short period super-lattice. The growth continued with a tunnel junction (TJ) which consists of p^{++}-$Al_{0.35}Ga_{0.65}N$/n^{++}-GaN/n^{++}-$Al_{0.42}Ga_{0.58}N$ where the n^{++}-layers have a Si concentration of $5x10^{19}$ cm^{-3} and the p^{++}-layers a Mg concentration of about $7x10^{19}$ cm^{-3}. A thickness of the n^{++}-GaN interlayer of 0 and 4 nm was explored. After growing the p^{++}-AlGaN layer, the structure was in-situ thermally annealed under nitrogen to activate the Mg acceptors. Finally, a 220 nm thick n-$Al_{0.42}Ga_{0.58}N$ lateral current spreading layer was grown.

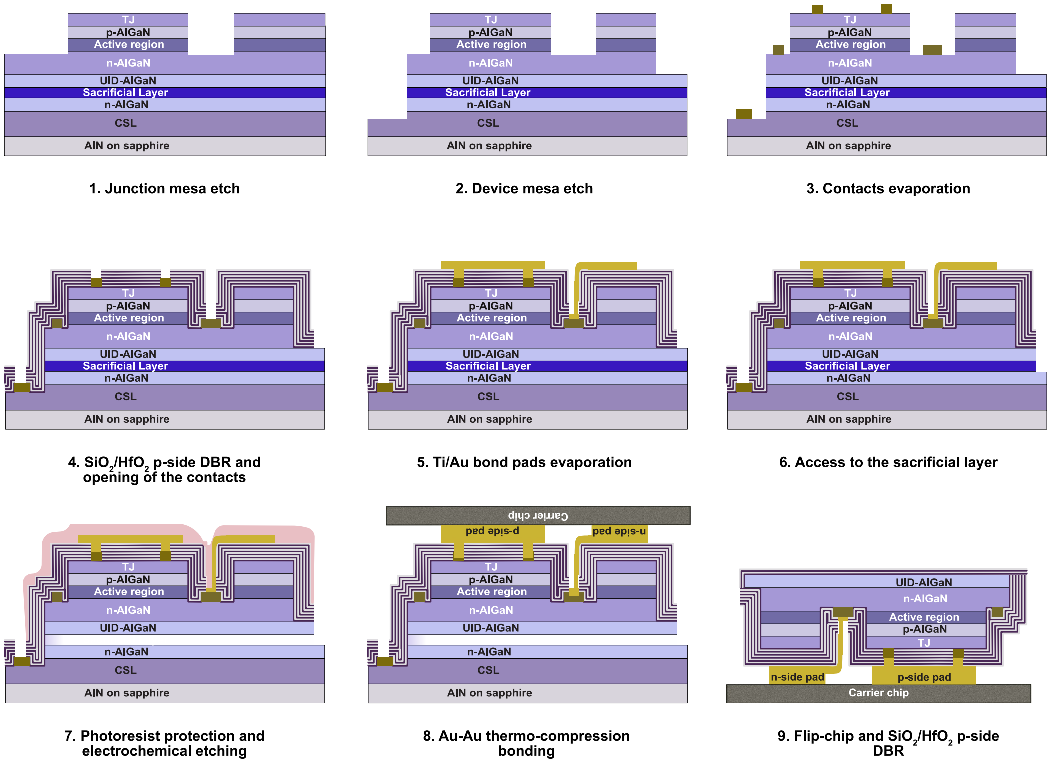

Figure S1: Schematic of the fabrication steps for realizing UVB RCLEDs.

Device fabrication

The UVB resonant-cavity LED (RCLED) fabrication, schematically displayed in Figure S1, started with the definition of a double mesa by two dry etch steps using a two-step chlorine-based inductively coupled plasma reactive ion etching (ICP-RIE)[6] process. The first mesa (circular active region mesa) gives access to the LED n-side and the second to the current spreading layer for electrochemical etching. The mesas defining the active region have diameters of 30 μm, 60 μm, 120 μm, and 160 μm. Because of the combination of the TJ with a top current spreading layer, the contacts to both the p- and n-side of the LED are on n-type material, thus a V(15 nm)/Al(90 nm)/Ni(20 nm)/Au(120 nm) stack could be used for both and deposited in a single e-beam evaporation and lift-off step. A short dip in buffered oxide etch was done prior to metal deposition. The annealing of the contacts and a second Mg activation were done in a single annealing step at 700 °C for 15 min in a nitrogen atmosphere by rapid thermal processing. The p-side contacts have a doughnut-shaped geometry, while

the n-side contacts surround the active region mesa. A 12-pair SiO_2/HfO_2 p-side distributed Bragg reflector (DBR) was sputtered with an aimed center wavelength at 310 nm. The p-side DBR, with a total thickness around ~1 μm, also acts as protection against parasitic electrochemical etching of the TJs and holds the LED to the substrate when the sacrificial layer is etched away. Subsequently, the bottom DBR was dried etched to expose the p- and n-side contacts by Ar/NF_3 ICP-RIE using an Al(10 nm)/Ni(200 nm) hard mask. Then, the hard mask was removed with NiCr etchant at 45 °C, and Ti(45 nm)/Au(600 nm) bond-pads were e-beam evaporated. Before carrying out the electrochemical etching, the devices were covered with a 5-μm thick positive photoresist for additional protection especially of the exposed metal contacts. The electrochemical etching was performed in a 0.3 M HNO_3 electrolyte with an applied bias voltage of 13 V for 22 min, see also.[2] After stripping the photoresist, the LED membranes were Au-Au thermo-compressibility bonded to a Si carrier chip with pre-defined Au metal pads.

Device characterization

Electro-optical characterization was performed by measuring the light-current-voltage (LIV) with a Hamamatsu S2281 Si photodiode, a Yokogawa 7651 current source, and a Keithley 2700 multimeter. Additionally, to map the near-field, far-field (over a range of ± 20° with respect to the cavity axis), and measure the electroluminescence (EL) a 750-mm-focal-length monochromator and 2048 x 512 pixel charge-couple device camera were employed. To realize temperature dependence EL, a PID-controlled resistive heater was mounted on the stage. The far-field emission pattern over a range of ± 80° was measured by a calibrated UV-enhanced Si-photodiode on a one-axis rotation stage. In Figure 2, the current density is estimated by assuming a homogeneous current injection over the whole area of the active region, while in Figure 3 and 4 the non-homogeneous current injection over the active region has been taken into account.

Devices with different TJ designs

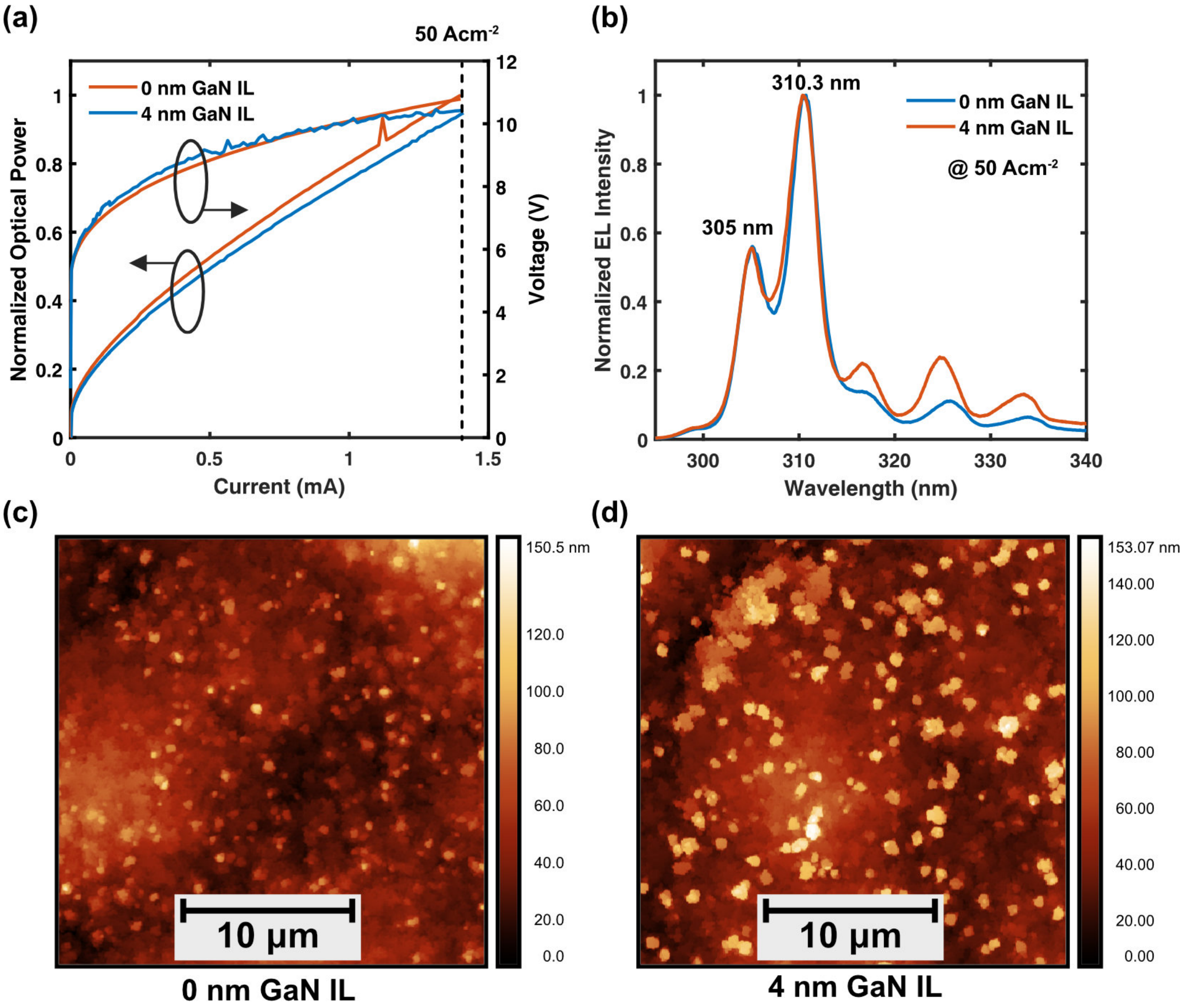

Figure S2: (a) LIV characteristics and (b) EL spectra of fully processed RCLEDs with different TJ designs. Atomic force microscope image of the as-grown surface of the samples with TJ including (c) a 0 nm and (d) a 4 nm GaN IL over a 25μm×25μm area.

Figure S2a and b show the electro-optical characterization of RCLEDs fabricated with a TJ including a 0 nm and 4 nm GaN interlayer (IL), where the emission originating above the p-side contacts has been removed from the DBR-RCLED in the EL spectra by filtering the spatially-resolved spectra. Both, the LIV characteristics (Figure S2a) and the EL spectra (Figure S2b) of the devices fabricated with the two different types of TJ do not show significant differences. On the other hand, Figure S2c and d show the rough as-grown surface of the epitaxial material with both TJ designs. The root mean square value is 16.45 nm and

21.5 nm for the sample with 0 nm and 4 nm GaN IL, respectively.

Devices with center disk-shaped p-side contact

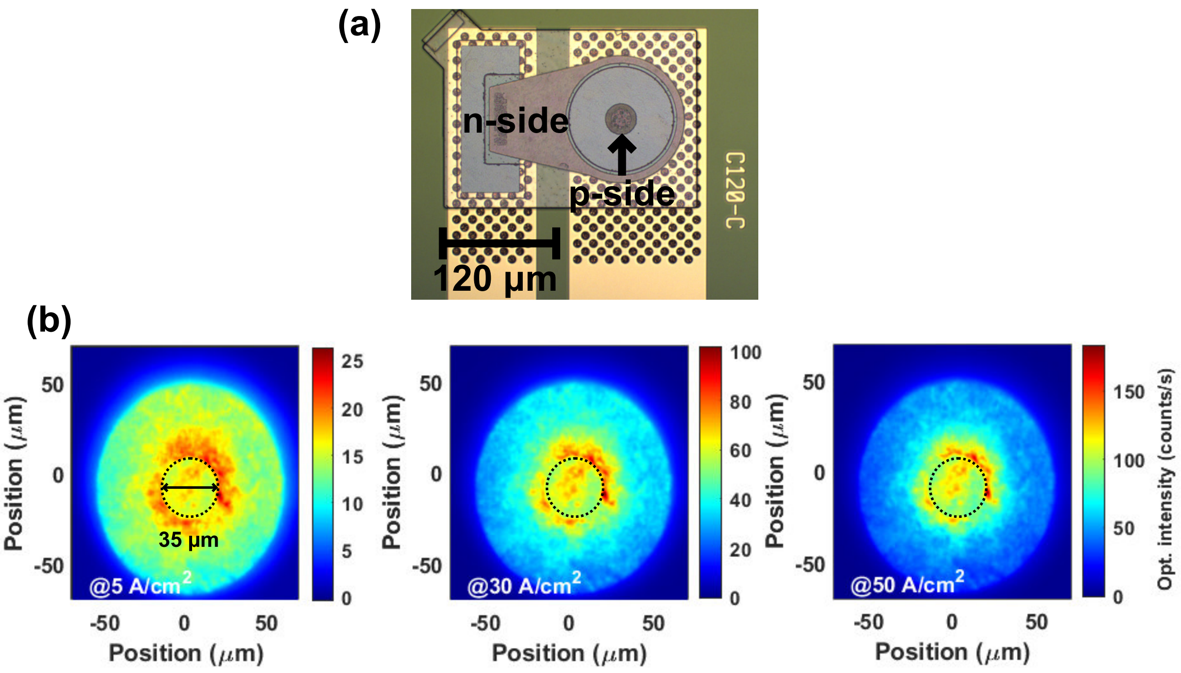

Figure S3: (a) Top view optical microscope image of a flip-chip RCLED with a mesa diameter of 120 μm and center disk-shaped p-side contact. (b) Near-field emission of the device driven at 5 A/cm^2, 30 A/cm^2, and 50 A/cm^2. The area delimited by the dashed lines corresponds to the border of the p-side contact

RCLEDs with a center disk-shaped p-side contact and an active region mesa diameter of 120 μm were fabricated and characterized for studying the p-AlGaN activation of the TJ, see in Figure S3a. The near-field emission of the device shows a stronger spontaneous emission proceeding from the periphery of the p-side contact when driven at 5 A/cm^2, 30 A/cm^2, and 50 A/cm^2 (Figure S3b) indicating current spreading limitation due to the thin top n-AlGaN current spreading layer. The intensity above the p-side contact is reduced due to the lower reflectivity of the contact material. Also, we conclude an effective activation of the p-AlGaN in the center of the mesa as the thermal activation during the device fabrication proceeds from the outer mesa edge to the center of the RCLEDs[7] and an incomplete activation in the center would lead to a brighter emission from the outer device mesa.

References

(1) Knauer, A.; Kolbe, T.; Rass, J.; Cho, H. K.; Netzel, C.; Hagedorn, S.; Lobo-Ploch, N.; Ruschel, J.; Glaab, J.; Einfeldt, S.; Weyers, M. High power UVB light emitting diodes with optimized n-AlGaN contact layers. *Japanese Journal of Applied Physics* **2019**, *58*, SCCC02.

(2) Bergmann, M. A.; Enslin, J.; Yapparov, R.; Hjort, F.; Wickman, B.; Marcinkevičius, S.; Wernicke, T.; Kneissl, M.; Haglund, Electrochemical etching of AlGaN for the realization of thin-film devices. *Applied Physics Letters* **2019**, *115*, 182103.

(3) Cardinali, G.; Hjort, F.; Prokop, N.; Enslin, J.; Cobet, M.; Bergmann, M. A.; Gustavsson, J.; Ciers, J.; Häusler, I.; Kolbe, T.; Wernicke, T.; Haglund, ; Kneissl, M. Low-threshold AlGaN-based UVB VCSELs enabled by post-growth cavity detuning. *Applied Physics Letters* **2022**, *121*, 103501.

(4) Bergmann, M. A.; Enslin, J.; Guttmann, M.; Sulmoni, L.; Ploch, N. L.; Hjort, F.; Kolbe, T.; Wernicke, T.; Kneissl, M.; Haglund, Increased Light Extraction of Thin-Film Flip-Chip UVB LEDs by Surface Texturing. *ACS Photonics* **2023**, *10*, 368.

(5) Enslin, J.-T. MOVPE growth of (In)AlGaN-based heterostructures for thin-film LEDs and VCSELs in the UVB spectral range. Ph.D. thesis, TU Berlin, 2021.

(6) Cho, H. K.; Kang, J. H.; Sulmoni, L.; Kunkel, K.; Rass, J.; Susilo, N.; Wernicke, T.; Einfeldt, S.; Kneissl, M. Low resistance n-contact for UVC LEDs by a two-step plasma etching process. *Semiconductor Science and Technology* **2020**, *35*, 095019.

(7) Kuwano, Y.; Kaga, M.; Morita, T.; Yamashita, K.; Yagi, K.; Iwaya, M.; Takeuchi, T.; Kamiyama, S.; Akasaki, I. Lateral Hydrogen Diffusion at p-GaN Layers in Nitride-Based Light Emitting Diodes with Tunnel Junctions. *Japanese Journal of Applied Physics* **2013**, *52*, 08JK12.
